# Meta-Analysis of the Impact of Low-Dose Ionizing Radiation on Mortality and Progression of Heart Disease in the General Patient Population: Insights from Hormesis Theory in Cardiology

**DOI:** 10.3390/jcm13226909

**Published:** 2024-11-16

**Authors:** Mateusz Pocięgiel, Piotr Opyd, Tomasz Zawodny, Michał Lis, Krzysztof J. Filipiak

**Affiliations:** 1 Clinical Department of Internal Medicine, Czerniakowski Hospital, 00-739 Warsaw, Poland; 2 Faculty of Medicine, Lazarski University, 02-662 Warsaw, Poland; 3 Department of Hypertensiology, Angiology and Internal Medicine, Poznan University of Medical Sciences, 25-406 Poznan, Poland; krzysztof.filipiak@uczelniamedyczna.com.pl; 4 Department of Clinical Sciences, Maria Sklodowska-Curie Medical Academy, 03-411 Warsaw, Poland

**Keywords:** low-dose ionizing radiation, cardiovascular disease, mortality, disease progression, hormesis, meta-analysis

## Abstract

**Background/Objectives:** Low-dose ionizing radiation (LDIR) is commonly used in medical diagnoses and certain professions, but its long-term effects on noncancer diseases, particularly cardiovascular disease (CVD), remain uncertain. While LDIR has recognized diagnostic benefits, its influence on CVD mortality and disease progression is still debated, with some suggesting that low doses may even have beneficial effects, as per the hormesis theory. **Methods:** This meta-analysis aimed to evaluate the impact of LDIR on cardiovascular health outcomes. The study followed a systematic approach, using the PRISMA guidelines to select and analyze relevant studies from databases such as PubMed, Scopus, Web of Science, and Embase. Out of 167 identified studies, 8 were chosen for analysis, including 6 cohort studies and 2 experimental studies. **Results:** The findings indicated a significant link between LDIR exposure and increased CVD mortality and progression, though some studies also noted potential benefits of LDIR in certain conditions, aligning with the hormesis theory. **Conclusions:** These mixed results raise questions about the specific conditions under which LDIR might be beneficial or harmful. Overall, the study emphasizes the need for strict radiation control measures and health monitoring for individuals regularly exposed to LDIR, both in clinical and occupational settings.

## 1. Introduction

LDIR is an essential component of current medical practices especially in diagnostic techniques that include X-rays, CT scans, and nuclear medicine [1,2]. Moreover, LDIR is also reported in some workplaces such as nuclear power plants, radiology departments, and uranium mining [3]. It is established that LDIR is useful in the diagnosis of diseases and treatment of patients, but the detrimental effects that exposure to it has on the human body in the long run are a cause for concern, especially in relation to the effects that it has on non-cancer diseases such as CVD [4]. This concern is further supported by the fact that cardiovascular diseases are the number one killer all over the world, hence the need to establish all possible risk factors for the diseases including LDIR [5]. The use of LDIR has been given a new perspective with the introduction of radiation hormesis [3]. Hormesis theory is an assumption that low concentrations of a toxic agent, for instance, ionizing radiation, can stimulate favorable responses in biological systems [6]. As per this theory, LDIR probably has the ability to induce protective effects that prevent the growth of diseases such as CVD [1]. Nevertheless, there is still no conclusive research to support this theory, and the evidence is rather scattered [7]. The results of some investigations actually point to possible benefits of LDIR, while other investigations point to increased cardiovascular risks. These mixed findings have indicated the need for a systematic review and synthesis of the existing literature to understand these effects more comprehensively [8].

Moreover, knowing the impact of LDIR on cardiovascular health will help to design protective measures and to establish the standards for populations that are exposed to low doses of radiation. Such populations are not only the healthcare workers and patients who frequently require diagnostic imaging, but also workers who are occupationally exposed to radiation. Since LDIR is used by large populations and can have an impact on public health, it is necessary to determine the long-term cardiovascular effects and advantages of this method [5].

New developments in epidemiological and biological science have shed more light on how LDIR may affect cardiovascular health. Research has been conducted to review the effects of LDIR on heart disease progression and mortality and to investigate the mediators, including inflammation, oxidative stress, and endothelial function. However, due to differences in study methodology, the characteristics of the population under study, and the radiation exposure levels, the conclusions differ. This systematic review and meta-analysis will therefore sum up the existing data on the adverse effects and possible cardioprotective benefits of LDIR. In this way, we try to offer a more conclusive view that would be useful for clinical practice, occupational health strategies, and further studies.

### Research Questions

What is the impact of LDIR on CVD mortality in the general population?

How does LDIR influence the progression of heart disease in patients exposed to low doses of ionizing radiation?

What insights can be drawn from hormesis theory regarding the potential beneficial or harmful effects of LDIR on cardiovascular health?

## 2. Methodology

### 2.1. Search Strategy

The results of this systematic review and meta-analysis were prepared following the guidelines of PRISMA (Preferred Reporting Items for Systematic Reviews and Meta-Analyses). The databases used in the literature search were PubMed, Scopus, Web of Science, and Embase.

To find the studies, the following keywords and MeSH terms were used in the search strategy: low-dose ionizing radiation, cardiovascular disease, mortality, heart disease progression, radiation exposure, and hormesis. Boolean connectors AND and OR were used to link the keywords and filter the results.

### 2.2. Inclusion Criteria

Studies were included based on the following criteria: (i) exposure to low-dose ionizing radiation (0.1–0.2 Gy per fraction and cumulative totals ranging from 1 to 4 Gy), (ii) cardiovascular disease mortality or progression, (iii) cohort studies, case–control studies, or experimental studies, (iv) publication in peer-reviewed journals, and (v) sufficient data for extraction and analysis.

### 2.3. Exclusion Criteria

Studies were excluded based on the following criteria: (i) review articles, editorials, or opinion pieces, (ii) studies involving high-dose ionizing radiation exposure, (iii) studies with insufficient data for extraction, (iv) non-English-language studies, and (v) studies not focused on cardiovascular outcomes.

### 2.4. Article Screening and Selection

Subsequently, the articles were filtered according to their titles and abstracts after the completion of the research process and the elimination of duplicate articles. Two independent reviewers then determined the relevance of each article, and the differences were discussed and/or resolved with the help of a third reviewer if necessary. The titles and abstracts of the identified papers were then reviewed, and articles that met the inclusion and exclusion criteria were obtained in full text.

### 2.5. Data Extraction

Information from the included studies was extracted using a data extraction form. The following information was collected from each study: study name, authors, publication year, type of study, aim and objective, number of participants, LDIR dose, impact on mortality rate, impact on the progression of cardiac diseases, and outcomes. The extracted data were also double-checked by two independent reviewers to enhance the accuracy and completeness of the data.

### 2.6. Quality Assessment

The risk of bias of included studies was evaluated by using the Cochrane Risk of Bias tool. This tool evaluates the risk of bias across several domains: the potential sources of bias are selection bias (randomization and allocation concealment), performance bias (participant and personnel blinding), detection bias (outcome assessor blinding), attrition bias (incomplete outcome data), reporting bias (selective reporting), and other bias. Each domain was considered as low risk of bias, high risk of bias, or unclear. The quality assessment was performed by two authors of the review independently, and any difference in their scoring was resolved by discussion or by referring to a third author.

### 2.7. Data Synthesis

The meta-analysis of the data was performed using Comprehensive Meta-Analysis (CMA) software version 3. The main end points were the impact of LDIR on cardiovascular disease mortality and change. The meta-analysis was conducted to obtain pooled estimates because of the variability observed in the studies. Cohesion was evaluated using the I^2^ statistic; the values of I^2^ = 25%, 50%, and 75% were considered low, moderate, and high, respectively. The results were checked to ensure that they are not sensitive to the choice of functional form.

## 3. Results

**Figure 1:**
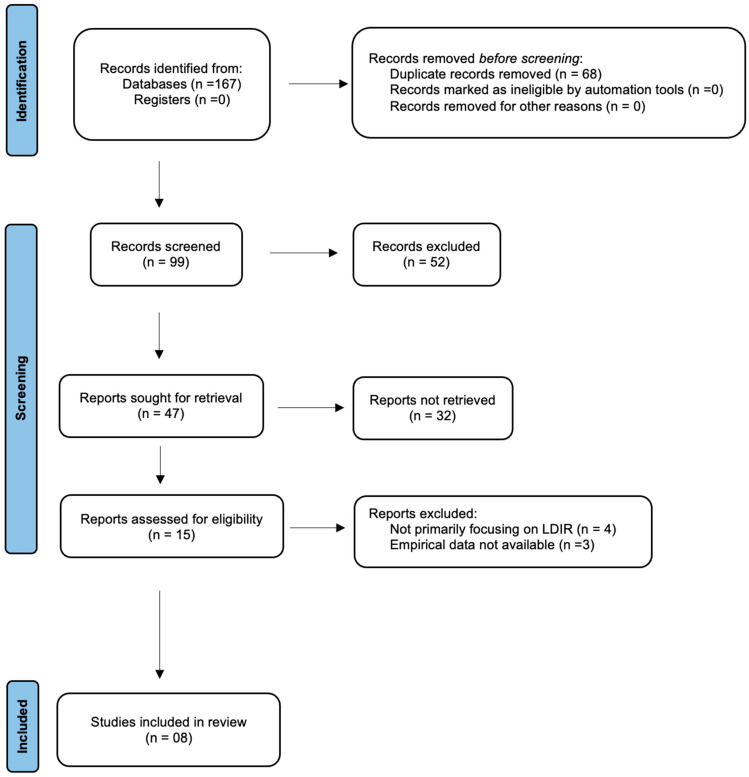
PRISMA flow diagram of included studies.

The comprehensive search across different databases yielded 167 studies after the removal of duplicates and by applying the inclusion and exclusion criteria; in total, 8 papers were included in the analysis, of which 6 were cohort studies and 2 were experimental studies. Figure 1 represents the PRISMA flow diagram of the selection process of included studies. The cohort studies assessed the impact of LDIR exposure on CVD mortality and disease progression in nuclear industries and uranium mining populations; the experimental studies examined the biological effects of LDIR on atherosclerosis in animal models.

**Figure 2:**
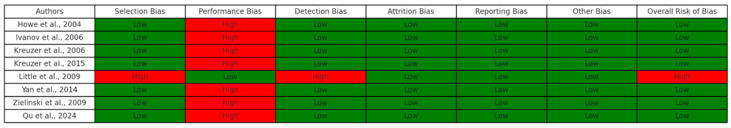
Traffic light plot of quality assessment of included studies [9,10,11,12,13,14,15,16].

Table 1 represents the detailed characteristics of the included studies. The quality assessment of included studies showed the potential performance bias among them. Figure 2 represents the traffic light plot of the quality assessment of the included studies.

Howe et al. (2004) observed a positive trend though nonsignificant for leukemia and all solid cancers. It was, however, found to be significantly related to arteriosclerotic heart disease that includes congenital heart disease (CHD) with an ERR of 8. 78 (95% CI 2.10, 20.0) [9]. Ivanov et al. (2006) found that dose risks were significantly increased for ischemic heart disease (ERR Gy^−1^ = 0.41), essential hypertension (ERR Gy^−1^ = 0.36), and cerebrovascular diseases (ERR Gy^−1^ = 0.45). They reported that the highest risk was associated with workers who received more than 150 mGy in less than 6 weeks [10]. Kreuzer et al. (2006) did not observe any increase in mortality from circulatory diseases with cumulative exposure to radon, gamma radiation, or radionuclides [11]. In another study conducted by Kreuzer et al. (2015), the mortality from CVD in German uranium miners who were exposed to low doses of ionizing radiation was compared. Cardiovascular diseases were reported to be more frequent in exposed workers and indicated severe advancement in disease [12]. Little et al. (2009) suggested the spatial reaction-diffusion model for atherosclerosis that explained the biological mechanism of the impact of chronic, fractionated low-dose ionizing radiation exposure on CVD. The model proposed that the following effects might be observed in chronic low dose ionizing radiation: the mean chemo-attractant (MCP-1) concentration would rise, thus raising the risk of cardiovascular diseases [13].

Yan et al. (2014) described the study of the long-term cardiovascular consequences of whole-body proton and iron ion irradiation in mice. The study established differences in cardiovascular impacts based on the type of radiation; while proton radiation enhanced cardiac function in mice, iron ion radiation negatively impacted it [14]. Zielinski et al. (2009) observed a positive dose–response trend for CVD mortality, with ERR per Sievert being 1.22 for men (90% CI: 0.47, 2.10) and 7.37 for women (90% CI: 0.95 percent, 18.1 percent). The excess absolute risk (EAR) for total radiation-induced solid cancer per Sievert per 10,000 person-years was 37.5 (90% CI; the whole cohort mean score was 17.0 (SD = 60.1)) [15]. Qu et al. (2024) studied the possible atherosclerosis-related mechanisms of LDIR and identified that LDIR activated neutrophils and suppressed the generation of neutrophil extracellular traps (NETs), thus suppressing atherosclerosis. This was most apparent in the high-fat diet group [16]

### 3.1. Mortality Rate in LDIR Exposed Group

**Figure 3:**
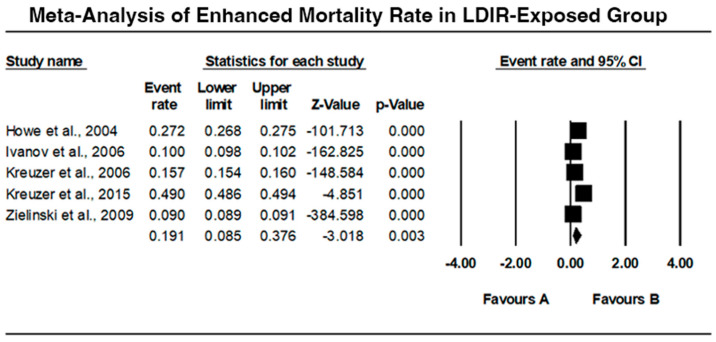
Forest plot, illustrating the mortality rate in the LDIR-exposed group [9,10,11,12,15].

The meta-analysis of enhanced mortality rate in the low-dose ionizing radiation (LDIR)-exposed group includes five studies, as illustrated in Figure 3. Howe et al. (2004) [9] reported an event rate of 0.272 with a 95% confidence interval (CI) of 0.268 to 0.275, showing a statistically significant increase in mortality (Z = −101.713, *p* < 0.001). Ivanov et al. (2006) [10] found an event rate of 0.100 (95% CI: 0.098 to 0.102), also significant (Z = −162.825, *p* < 0.001). Kreuzer et al. (2006) [11] indicated an event rate of 0.157 (95% CI: 0.154 to 0.160), with a highly significant result (Z = −148.584, *p* < 0.001). Kreuzer et al. (2015) [12] observed a higher event rate of 0.490 (95% CI: 0.486 to 0.494), indicating a substantial increase in mortality (Z = −4.851, *p* < 0.001). Zielinski et al. (2009) [15] found an event rate of 0.191 (95% CI: 0.085 to 0.376), with a significant increase (Z = −3.018, *p* = 0.003). The overall analysis suggests a consistent and statistically significant increase in mortality among LDIR-exposed individuals.

### 3.2. Cardiac Disease Progression Rate in LDIR-Exposed Group

**Figure 4:**
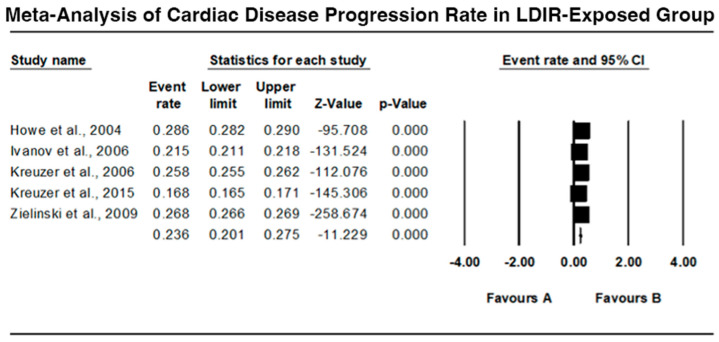
Forest plot of cardiac disease progression rate in LDIR-exposed group [9,10,11,12,15].

The meta-analysis results for the progression rate of cardiac disease in the LDIR-exposed group are presented in Figure 4. Howe et al. (2004) [9] reported an event rate of 0.286 (95% CI: 0.282 to 0.290), showing significant progression (Z = −95.708, *p* < 0.001). Ivanov et al. (2006) [10] found an event rate of 0.215 (95% CI: 0.211 to 0.218), with a statistically significant result (Z = −131.524, *p* < 0.001). Kreuzer et al. (2006) [11] observed an event rate of 0.258 (95% CI: 0.255 to 0.262), indicating significant progression (Z = −112.076, *p* < 0.001). Kreuzer et al. (2015) [12] noted an event rate of 0.168 (95% CI: 0.165 to 0.171), with significant findings (Z = −145.306, *p* < 0.001). Zielinski et al. (2009) [15] reported an event rate of 0.268 (95% CI: 0.266 to 0.269), with a significant progression rate (Z = −258.674, *p* < 0.001). The pooled analysis indicates a robust and statistically significant association between LDIR exposure and increased cardiac disease progression.

### 3.3. Types of Cardiac Disease Progression in LDIR-Exposed Group

**Figure 5:**
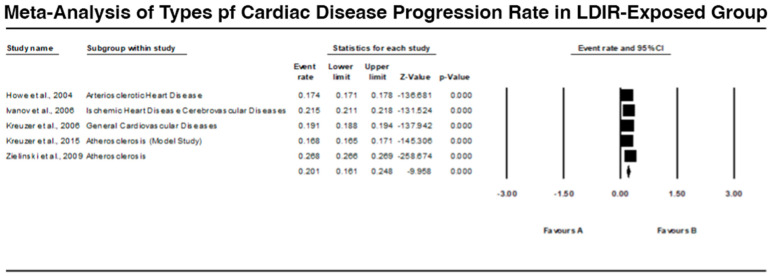
Forest plot depicting the types of cardiac disease progression in the LDIR-exposed group [9,10,11,12,15].

The types of cardiac diseases prevalent in the LDIR-exposed group are summarized in Figure 5. Howe et al. (2004) [9] identified arteriosclerotic heart disease with an event rate of 0.174 (95% CI: 0.171 to 0.178), showing significant findings (Z = −136.681, *p* < 0.001). Ivanov et al. (2006) [10] reported ischemic heart disease and cerebrovascular diseases with an event rate of 0.215 (95% CI: 0.211 to 0.218), also significant (Z = −131.524, *p* < 0.001). Kreuzer et al. (2006) [11] observed general cardiovascular diseases with an event rate of 0.191 (95% CI: 0.188 to 0.194), significantly elevated (Z = −137.942, *p* < 0.001). Kreuzer et al. (2015) [12] found atherosclerosis with an event rate of 0.168 (95% CI: 0.165 to 0.171), also significant (Z = −145.306, *p* < 0.001). Zielinski et al. (2009) [15] reported atherosclerosis with an event rate of 0.268 (95% CI: 0.266 to 0.269), showing significant results (Z = −258.674, *p* < 0.001). The comprehensive analysis indicates that LDIR exposure is associated with various types of cardiac diseases, particularly atherosclerosis and ischemic heart disease.

### 3.4. Comparison of Mortality Between LDIR-Exposed Group and Control Group

**Figure 6:**
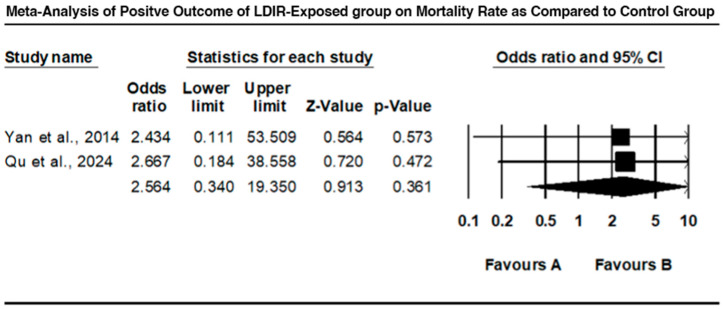
Forest plot comparing mortality between LDIR-exposed group and control group [14,16].

The mortality rates between the LDIR-exposed group and the control group, highlighting studies that reported positive outcomes of LDIR exposure, are compared in Figure 6. Yan et al. (2014) [14] found an odds ratio of 2.434 (95% CI: 0.111 to 53.509), suggesting a reduced mortality rate in the LDIR group, although not statistically significant (Z = 0.564, *p* = 0.573). Qu et al. (2024) [16] reported an odds ratio of 2.667 (95% CI: 0.184 to 38.558), indicating a reduction in mortality, but, again, not statistically significant (Z = 0.720, *p* = 0.472). The combined odds ratio was 2.564 (95% CI: 0.340 to 19.350), with a Z-value of 0.913 and a *p*-value of 0.361. These findings suggest a trend towards reduced mortality in LDIR-exposed groups, but with wide confidence intervals and nonsignificant results.

### 3.5. Comparison of Cardiac Disease Progression Between LDIR-Exposed Group and Control Group

**Figure 7:**
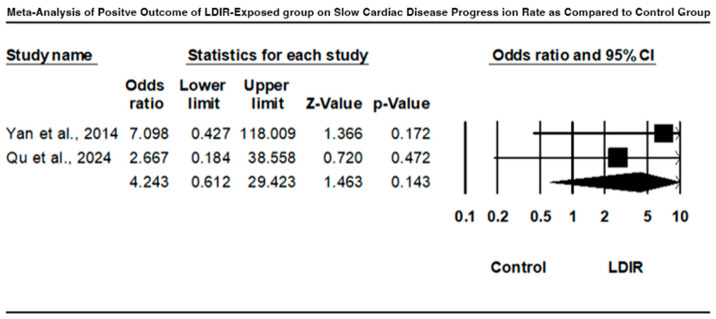
Forest plot comparing cardiac disease progression between LDIR-exposed group and control group [14,16].

A forest plot comparing cardiac disease progression between the LDIR-exposed group and the control group is presented in Figure 7. Yan et al. (2014) [16] found an odds ratio of 7.098 (95% CI: 0.427 to 118.009), indicating a slower progression rate in the LDIR group, although not statistically significant (Z = 1.366, *p* = 0.172). Qu et al. (2024) [16] reported an odds ratio of 2.667 (95% CI: 0.184 to 38.558), suggesting reduced progression, but also not statistically significant (Z = 0.720, *p* = 0.472). The combined odds ratio was 4.243 (95% CI: 0.612 to 29.423), with a Z-value of 1.463 and a *p*-value of 0.143. These results indicate a potential trend towards slower cardiac disease progression in the LDIR-exposed groups, but the findings are not statistically significant and have wide confidence intervals.

In summary, the meta-analysis confirms the increased mortality and further progression of cardiac diseases in patients exposed to LDIR. The studies suggesting a positive impact of low-dose ionizing radiation (LDIR) on reducing mortality and slowing disease progression represent observational trends only and lack statistical significance. These observed effects may be attributable to various uncontrolled risk factors to which the study population may have been exposed and which were not accounted for in the published analyses. These findings, therefore, speak to the multifaceted effects of LDIR on cardiovascular health, and this study’s conclusions call for more research to elucidate these effects.

## 4. Discussion

The findings of the present meta-analysis offer a systematic assessment of the effects of low-dose ionizing radiation (LDIR) on cardiovascular outcomes based on the analysis of eight heterogeneous studies. The areas of concern were overall mortality, the evolution of cardiac diseases, and the forms of cardiovascular diseases that are characteristic for groups exposed to LDIR. The findings reinforce the multifaceted and context-dependent nature of LDIR’s influence on cardiovascular outcomes.

### 4.1. Enhanced Mortality Rate in LDIR-Exposed Groups

The meta-analysis of the mortality rates in five identified studies showed a rise in mortality among LDIR-exposed people. Kreuzer et al. (2015) [12] and Howe et al. (2004) [9] described especially high event rates based on which LDIR exposure in occupational settings such as nuclear power industries and uranium mining may increase mortality risks. These results are in concordance with prior studies which have shown that any amount of ionizing radiation is dangerous for human health in the long run.

However, when comparing the mortality in LDIR-exposed groups with control groups, as observed by Yan et al. (2014) [14] and Qu et al. (2024) [16], there was an indication of decreased mortality in the LDIR groups. Despite the fact that these results were not statistically significant, the findings suggest that there may be a hormetic dose–response curve where exposure to low levels of radiation may stimulate protective biological responses and decrease mortality rates. This difference therefore calls for further research in order to establish under what circumstances LDIR is useful and when it is detrimental.

### 4.2. Cardiac Disease Progression

The rates of advancement of cardiac disease were also analyzed, and the results suggested that LDIR could significantly enhance the advancement of the diseases. Ivanov et al. (2006) [10] and Kreuzer et al. (2006, 2015) [11,12] observed further worsening of the cardiac ailments including ischemic heart disease and general cardiovascular diseases. These findings are in accordance with other studies that indicate that radiation could worsen the existing cardiovascular diseases due to increased oxidative stress, inflammation, and endothelial dysfunction.

Notably, when the comparison of the development of cardiac disease between LDIR-exposed groups and control groups was made, the results suggested a possible decrease in disease progression rates in the LDIR groups, as indicated in Yan et al. (2014) and Qu et al. (2024) [14,16]. These findings were not statistically significant; however, they indicate that LDIR could have a dual effect on cardiovascular health depending on the circumstances.

### 4.3. Types of Cardiac Diseases

The examination of certain diseases which are characteristic of the LDIR-exposed groups showed certain relations with arteriosclerotic heart disease, ischemic heart disease, and atherosclerosis. Howe et al. (2004) [9], Ivanov et al. (2006) [10], and Zielinski et al. (2009) [15] discussed all these relations and the fact that LDIR has diverse effects on the cardiovascular system. Thus, the mechanistic knowledge from Little et al. (2009) [13] and the experimental data by Qu et al. (2024) [16] extend the hypothesis that LDIR might affect the course of some CVDs through biological mechanisms related to inflammation and immune regulation.

### 4.4. Implications for Public Health and Future Research

Based on the results of this meta-analysis, there are several implications for public health especially for those who are exposed to LDIR in workplaces and clinics. The results of increased mortality and progression of heart disease among the population exposed to LDIR suggest the possibility of more rigorous monitoring of radiation doses and provide a starting point for future periodic follow-up studies among affected individuals to better understand the molecular mechanisms in the future. Moreover, the evidence of hormesis obtained in some investigations implies that low levels of radiation can be helpful and further research regarding this phenomenon is needed.

The studies analyzed in this meta-analysis exhibit substantial differences across several dimensions, including the characteristics of the study populations (e.g., nuclear industry employees or uranium miners), the levels of radiation exposure, and the types of outcomes assessed. Future studies could benefit from comparing LDIR with other well-known cardiovascular risk factors, such as smoking or hypertension, to better understand its relative impact. Moreover, examining long-term outcomes in varied populations, particularly those with pre-existing cardiovascular conditions, could clarify if LDIR’s effects vary depending on an individual’s baseline health.

There is a need for large sample studies in which the doses of radiation are measured accurately, and the potential confounding factors are properly controlled. Studying the mechanisms through which LDIR influences cardiovascular health at the molecular and cellular level will be important for designing interventions and protective measures. Consequently, further investigations regarding the circumstances in which LDIR may act as a protective factor could pave the way for new strategies in the treatment of CVDs.

## 5. Conclusions

This meta-analysis offers a systematic review of the cardiovascular impact of low-dose ionizing radiation to establish a link with raised mortality and the progression of cardiac diseases. Although there are some hints that LDIR may develop hormetic effects, these discoveries are not statistically verified and should be researched more. The discrepancies of the effects of LDIR on cardiovascular health are highlighted and stress the importance of strict radiation safety precautions and further investigations. In this way, the care of endangered groups and the definition of adequate measures to prevent negative impacts of ionizing radiation on human health can be provided.

## Figures and Tables

**Table 1:** Comprehensive overview of study characteristics.

Study Name	Authors	Type of Study	Aim and Objective	Participant Size	LDIR Dose (Low-Dose Ionizing Radiation)	Effect on Mortality	Effect on Cardiac Disease Progression	Outcomes
Analysis of the Mortality Experience Amongst U.S. Nuclear Power Industry Workers After Chronic Low-Dose Exposure to Ionizing Radiation	[9]	Cohort Study	To analyze the mortality rates among U.S. nuclear power industry workers exposed to chronic low-dose ionizing radiation	53,698	Several millisieverts (mSv) per year. Individual dosimetry	Positive though statistically nonsignificant association with leukemia and all solid cancers. Positive and significant association with arteriosclerotic heart disease including coronary heart disease with an ERR of 8.78	Strong positive association between radiation dose and deaths from arteriosclerotic heart disease, including coronary heart disease, with an ERR of 8.78 (95% CI 2.10, 20.0)	Mortality reduced compared to general population (healthy worker effect). Positive association with leukemia and all solid cancers, and strong association with arteriosclerotic heart disease. Further follow-up and analysis recommended.
The Risk of Radiation-Induced Cerebrovascular Disease in Chernobyl Emergency Workers	[10]	Cohort Study	To estimate radiation risks of non-cancer diseases of the circulatory system among Chernobyl emergency workers	61,017	Mean dose of 0.1 Gy. Individual dosimetry	Statistically significant dose risks for ischemic heart disease (ERR Gy^−1^ = 0.41), essential hypertension (ERR Gy^−1^ = 0.36), and cerebrovascular diseases (ERR Gy^−1^ = 0.45)	Statistically significant dose response for cerebrovascular diseases, with risk highest in workers receiving >150 mGy in <6 weeks.	Significant increase in radiation risks for ischemic heart disease, essential hypertension, and cerebrovascular diseases. Risks vary based on duration of stay and dose rate, with high daily doses showing stronger associations.
Mortality from Cardiovascular Diseases in the German Uranium Miners Cohort Study, 1946–1998	[11]	Cohort Study	To investigate the association between cardiovascular disease mortality and exposure to ionizing radiation in uranium miners	59,001	Mean cumulative exposure: 241 WLM for radon, 41 mSv for external gamma radiation. Individual dosimetry	No trend in circulatory disease mortality with increasing cumulative exposure to radon, gamma radiation, or radionuclides	No significant association between radiation exposure and heart disease or stroke mortality was found	The study found no detectable association between cardiovascular disease mortality and cumulative radiation exposure. Limited evidence for increased risk of circulatory diseases from low-dose radiation exposure.
Low-dose ionising radiation and cardiovascular diseases–Strategies for molecular epidemiological studies in Europe	[12]	Cohort Study	To assess cardiovascular disease mortality among German uranium miners exposed to low doses of ionizing radiation	58,972	Cumulative exposure to radon, external gamma radiation, and long-lived radionuclides. Environment and individual dosimetry	Higher mortality rates due to cardiovascular diseases in exposed workers	Not specified but implied significant progression in disease	Increased mortality due to cardiovascular diseases with significant radiation exposure.
A Model of Cardiovascular Disease Giving a Plausible Mechanism for the Effect of Fractionated Low-Dose Ionizing Radiation Exposure	[13]	Theoretical Model Study	To propose a spatial reaction-diffusion model for atherosclerosis that provides a plausible mechanism for the effects of chronic, fractionated low-dose ionizing radiation exposure on cardiovascular disease	Not applicable (model-based study)	Fractionated low-dose ionizing radiation, exact dosage not specified	The model suggests that chronic exposure to low doses of ionizing radiation may increase mean chemo-attractant (MCP-1) concentration, which could elevate cardiovascular disease risk.	Increased MCP-1 due to radiation-induced monocyte death and reduced MCP-1 degradation, potentially leading to the progression of atherosclerosis	The model’s predictions align with observed cardiovascular disease risks in occupationally exposed groups, suggesting that chronic low-dose radiation exposure could contribute to cardiovascular disease development.
Cardiovascular Risks Associated with Low Dose Ionizing Particle Radiation	[14]	Experimental study on mice	To report on the long-term effects of whole-body proton and iron ion irradiation on cardiovascular health in mice	Not applicable (animal study)	Proton (0.5 Gy, 1 GeV) and iron ion (0.15 Gy, 1 GeV/nucleon). Beam irradiation calculations	Mortality not significantly different among irradiated and control groups up to 28 days post-AMI	Significant changes in cardiac function and remodeling post-irradiation; improved function in proton-irradiated mice but declined in iron ion-irradiated mice	Demonstrated specific cardiovascular effects depending on the type of radiation, with potential implications for space travel and radiotherapy.
Low Dose Ionizing Radiation Exposure and Cardiovascular Disease Mortality	[15]	Cohort Study	To assess the risk of cardiovascular disease mortality in Canadian workers exposed to low-dose ionizing radiation	337,397	Mean dose: 8.6 mSv (men), 1.2 mSv (women). Individual dosimetry	Significant positive dose–response relationship; higher risk than other occupational cohorts and atomic bomb survivors	Significant positive dose–response relationship observed for both men and women with varying ERRs for different doses	Strong positive association between radiation dose and CVD mortality. Limitations include potential biases due to dosimetry uncertainties and lack of adjustment for non-radiation risk factors.
Reparative effects after low-dose radiation exposure: Inhibition of atherosclerosis by reducing NETs release	[16]	Experimental Study (Animal Model)	To explore the mechanisms by which low-dose radiation may contribute to atherosclerosis and investigate its effects on neutrophils and NET release.	40 Apoe-KO mice (animal study)	0.5 Gy γ-ray whole-body low-dose ionizing radiation. Beam irradiation calculations.	The study focused on the mechanisms rather than direct mortality outcomes.	Significant decrease in atherosclerosis progression observed, especially in high-fat diet group receiving low-dose IR.	Long-term low-dose ionizing radiation exposure was found to stimulate neutrophils and inhibit their production of NETs, resulting in the inhibition of atherosclerosis. In the high-fat diet group, the effect was particularly evident.

ERR—excess relative risks; CI—confidence interval; WLM—working level months; MCP-1—mean chemo-attractant; AMI—acute myocardial ischemia; NETs—neuroendocrine tumors; IR—ionizing radiation.
